# A Case of Neuroleptic Malignant Syndrome in Pregnancy

**DOI:** 10.7759/cureus.4211

**Published:** 2019-03-09

**Authors:** Maanvi Mittal, Phoebe Garcia, Jason P Lee, Davin Agustines

**Affiliations:** 1 Psychiatry, Western University of Health Sciences, Pomona, USA; 2 Miscellaneous, University of California Los Angeles, Los Angeles, USA; 3 Psychiatry, Olive View - University of California Los Angeles Medical Center, Los Angeles, USA

**Keywords:** neuroleptic malignant syndrome, pregnancy, antipsychotics, peripartum, nms, psychosis, extrapyramidal symptoms, eps, seroquel®, haldol®

## Abstract

Neuroleptic malignant syndrome (NMS) is an obstetric emergency. Management of acute psychosis in a pregnant patient remains complicated with limited therapeutic options due to teratogenic effects. We report a case in which a patient’s antipsychotic regimen during a viable pregnancy subsequently led to NMS, and discuss our chosen management for treating NMS.

## Introduction

Neuroleptic malignant syndrome (NMS), a complication that can result from antipsychotic use, is considered an emergency in pregnancy due to the accompanying symptoms of hyperthermia, mental-status changes, muscle rigidity and autonomic instability [1]. Furthermore, potential complications of NMS place the patient at risk for rhabdomyolysis, renal failure, and arrhythmias. The most current, accepted pathophysiology of NMS in a psychiatric setting is dopamine receptor blockade from antipsychotics, and an increased release of calcium from skeletal sarcoplasmic reticulum [2].

In peripartum patients, the potential options for treatment of psychosis require a careful analysis. The American College of Obstetrics and Gynecology has clinical guidelines suggesting typical antipsychotics have a safer profile than atypical antipsychotics in pregnant women [3]. However, atypical antipsychotics have a lower risk of extrapyramidal symptoms and NMS than typical antipsychotics.

The diagnosis and management of NMS also present as difficult decisions in the acute inpatient setting. NMS mimics many other conditions, such as extrapyramidal symptoms, catatonia, encephalopathy, hyperthyroidism and allergic reactions. When the condition arises and is correctly identified in pregnancy, the various treatment options have to be considered carefully for teratogenic effects and fetal toxicity.

Pregnant patients on antipsychotics should be closely monitored for NMS, as it is a medical emergency and can rapidly become fatal. This case report seeks to support and enhance the current literature on NMS in pregnant patients in order to highlight a unique patient presentation and aid in future diagnosis.

## Case presentation

A 19-year-old female, with a history of two pregnancies, neither of which reached a gestational age of 24 weeks, a current viable intrauterine pregnancy at 32 weeks and past medical history of recently diagnosed unspecified psychosis, methamphetamine use disorder and history of multiple incarcerations, was brought in by ambulance to our psychiatric emergency room from a women’s correctional facility. She presented with persistent delusions, paranoia and persecutory thought content related to her pregnancy.

During her psychiatric evaluation in the emergency department, the patient reported having auditory and visual hallucinations. She was observed responding to internal stimuli, displayed disorganized thought process, and was notably agitated, all in the context of primary psychosis versus drug-induced psychosis. She had been prescribed 300 mg of quetiapine daily before this hospitalization, but was unable to confirm or deny whether she had been taking the medication as prescribed.

At the start of this hospitalization, she was given quetiapine 300 mg twice daily for three days. She also received, as needed, a 3-mg intramuscular dose of haloperidol for acute agitation and violent behavior.

Her prescribed quetiapine dose was subsequently increased to 400 mg twice daily for another four days, but her ongoing aggression resulted in an additional intramuscular dose of 5 mg of haloperidol.

Because of poor response on this antipsychotic regimen, with continued auditory hallucinations and psychotic agitation, the patient was cross-titrated from quetiapine 400 mg twice daily to haloperidol 5 mg twice daily. During this time, she attempted to elope from the unit on three separate occasions due to persecutory delusions. She also continued to exhibit disorganized and aggressive behaviors towards staff, but no restraints were used. In addition, the patient began to develop sialorrhea and mild tremors. Due to the onset of these symptoms, haloperidol dosing was reduced to 5 mg once daily. The patient was also started on diphenhydramine 25 mg daily for her mild tremors, sialorrhea, and muscle rigidity, which were initially thought to be extrapyramidal symptoms caused by haloperidol. Labs were drawn and indicated a white blood cell count (WBC) on the upper limits of normal (10.8). Her basic metabolic panel (BMP) was notable for Na of 132, aspartate transaminase (AST) of 50, lactate dehydrogenase of 213 and creatine kinase of 1029. She was afebrile, but tachycardic with a pulse as high as 119.

The patient continued to be monitored, but remained aggressive and violent towards staff and received an additional 3 mg intramuscular dose of haloperidol. Her muscle rigidity persisted and she began complaining of worsening stiffness.

On hospital day 21, her care was transferred to the inpatient medicine team due to increased clinical suspicion for NMS. Her vitals remained stable. Labs were redrawn and showed an uptrending WBC of 11.5, partial thromboplastin time (PTT) of 440’s, D-dimer of 2640, Na of 133, alkaline phosphatase of 186, AST of 76, lactate dehydrogenase of 265 and uptrending creatine kinase at 2,320. She was given supportive care with 100 cc/hr intravenous normal saline maintenance and was monitored for autonomic instability including tachycardia, arrhythmia, and blood pressure lability, which are known to characterize NMS [1].

Her labs were then redrawn after normal saline infusion and indicated uptrending creatine kinase to 2,405, lactate dehydrogenase to 272, AST to 79 and alkaline phosphatase to 189 along with tachycardia and otherwise stable vitals.

Five days after being transferred to inpatient medicine, she was admitted back to the psych inpatient unit with stable vitals and a down trending creatine kinase (CK) at 940. Her muscle rigidity, sialorrhea and tachycardia had also resolved. Upon transfer back to the inpatient psychiatric ward, all antipsychotics were stopped and she was started on 1 mg of lorazepam as needed for agitation.

At 39 weeks gestation she was transferred to the inpatient obstetrics unit for induction of labor. She was given 1 mg of lorazepam for agitation during delivery. She had a normal, spontaneous vaginal delivery of a baby boy without complications. Two days after delivery, her psychiatric hold allowing her to be detained in a hospital setting expired. She was discharged into a transitional care setting, with close outpatient follow-up. An antipsychotic regimen was not restarted at the time of discharge due to ongoing concerns that NMS had not resolved for at least two weeks prior to the rechallenge process.

## Discussion

While NMS has been well studied, the literature on its occurrence and presentation in pregnant patients is scarce. Of the six case reports on NMS occurring in the peripartum period, four cases involved haloperidol, either alone or in combination with another antipsychotic [4]. Ours is the first involving use of quetiapine, followed by haloperidol. This case not only contributes to the existing literature on NMS after haloperidol use, but also introduces an atypical presentation and discusses the importance of considering the development of NMS in the context of complex psychiatric and medical issues.

The American College of Obstetrics and Gynecology has clinical guidelines that suggest typical antipsychotics have a safer profile than atypical antipsychotics in pregnant patients; no teratogenic effects have been documented with chlorpromazine, haloperidol, or perphenazine [3]. This finding alone places pregnant patients at greater risk of developing NMS. Our patient was switched to haloperidol from quetiapine, and while her psychosis initially improved, she remained agitated on the unit.

Changes in dosage and intramuscular administration of antipsychotics have also been associated with an increased risk of NMS [5]. Our patient began exhibiting symptoms of NMS approximately 48 hours after switching from oral quetiapine to oral haloperidol, and after receiving an intramuscular haloperidol injection in addition to oral haloperidol. This suggests a need for heightened monitoring of pregnant patients should they receive intramuscular haloperidol administration, as further evidenced by the Escobar-Vidarte study, where symptoms presented 24 hours after administration of intramuscular haloperidol [4].

Furthermore, this patient’s presentation was atypical and emphasizes the difficulty of diagnosis, especially when the patient has a documented history of psychosis. The Diagnostic and Statistical Manual of Mental Disorders, 5th Edition criteria for diagnosis of NMS, requires meeting three major criteria and two minor criteria. The three major criteria include: exposure to dopamine blocking agent, severe muscle rigidity, and fever. The minor criteria include: diaphoresis, dysphagia, tremor, incontinence, altered level of consciousness, mutism, tachycardia, elevated or labile blood pressure, leukocytosis, and elevated levels of creatine phosphokinase [6]. Of the major criteria, our patient was on haloperidol and had notable physical examination findings including muscle rigidity; of the minor criteria, she initially presented with tremor, tachycardia, and mutism. A unique symptom of her NMS was sialorrhea, which has rarely been reported [2].

In regards to the major criteria of fever, hyperpyrexia can remain absent until 24 hours after initial presentation of symptoms, but our patient continuously remained afebrile [2]. In contrast to our report, three cases of haloperidol-associated NMS in pregnancy eventually developed a fever [4, 7-8].

Because our patient did not meet all major criteria, it was unclear to the treatment team if she was presenting with continued psychosis, an adverse reaction to medication, or the onset of NMS itself. Additionally, though her initial labs were normal, subsequent labs showed characteristic leukocytosis, transaminitis, and uptrending lactate dehydrogenase and creatinine kinase the following day [2]. Her abnormal presentation emphasizes the importance of continued monitoring of patients showing any symptoms of NMS before ruling out its diagnosis. At this point, consultations with obstetrics and medicine agreed on the diagnosis of NMS and the patient was transferred to the medicine team for management.

In our case, relatively early identification and prompt cessation of the antipsychotic was sufficient treatment. Our patient's vital signs, except for tachycardia, remained stable. Thus, dantrolene, the typical emergent antidote for NMS, was not considered. In contrast, James reported using bromocriptine for NMS in a pregnant patient due to its frequent use in obstetrics and studies demonstrating no harmful pregnancy outcome. Additionally, other reported treatments for NMS include benzodiazepines, dantrolene, amantadine, carbidopa/L-dopa, and propranolol. However, the use of these medications in pregnancy is rare and requires further investigation [9].

After the resolution of NMS, labor was induced at 39 weeks, due to the patient’s ongoing positive symptoms of psychosis, and worsening agitation. She experienced an uncomplicated vaginal delivery.

## Conclusions

As previous case reports have identified, the differential diagnosis for a pregnant patient presenting with symptoms of NMS is vast. It is important to be aware of the atypical presentations of NMS in pregnant patients and to continue monitoring objective lab measurements throughout the hospital course. This will allow for timely recognition and treatment of the syndrome, ensuring the safety of pregnant patients with psychosis.
